# Expression Patterns of T-Cell Intracellular Antigen 1 in Neurodegenerative Disorders and Stroke

**DOI:** 10.3390/ijms27052252

**Published:** 2026-02-27

**Authors:** Jiaqi Han, Hong Yu, Tianwen Zheng, Zhihui Huang, Lipei Wang

**Affiliations:** 1 School of Basic Medical Sciences, Hangzhou Normal University, Hangzhou 311121, China; 2024111027019@stu.hznu.edu.cn (J.H.); 2023111027014@stu.hznu.edu.cn (T.Z.); 2 School of Pharmacy, Hangzhou Normal University, Hangzhou 311121, China; 2023112025074@stu.hznu.edu.cn (H.Y.); huang0069@hznu.edu.cn (Z.H.)

**Keywords:** TIA1, stress granules, nervous system, stroke, neurodegenerative disorders

## Abstract

T-cell intracellular antigen 1 (TIA1) is a multifunctional RNA-binding protein (RBP) belonging to the RNA recognition motif (RRM) family. Under steady-state conditions, it is predominantly localized in the nucleus and highly expressed in the nervous system, where it regulates neuronal and glial functions. TIA1 modulates mRNA splicing, stability, and translation and promotes stress granule (SG) assembly under cellular stress. Recent studies indicate that the spatiotemporal dynamics of TIA1 in neurodegenerative contexts influence disease progression by regulating inflammatory responses, apoptosis, and related pathways. This review discusses the molecular structure and functions of TIA1, focusing on its expression in neurons and glia, as well as its implications in neurodegenerative disorders and stroke. The findings highlight TIA1 as a promising target for novel neuroprotective therapeutic strategies.

## 1. Introduction

In 1991, Paul Anderson published the seminal study that first identified TIA1 and described its role in T-cell-mediated cytotoxicity [1]. In the decades since, research has progressively underscored the importance of TIA1 in neurological disorders [2,3,4], particularly as its functional mechanisms in neurodegenerative diseases [5] and stroke have been further elucidated. Neurodegenerative diseases, a heterogeneous group of complex disorders characterized by the progressive loss or dysfunction of neurons in the central and peripheral nervous systems, are increasing in prevalence with global aging trends, presenting a serious worldwide public health challenge [6]. This category includes Alzheimer’s disease (AD), amyotrophic lateral sclerosis (ALS), multiple sclerosis (MS), Huntington’s disease (HD), and Parkinson’s disease (PD). Although clinically distinct, these neurodegenerative diseases share common pathogenic mechanisms, such as disrupted proteostasis, mitochondrial dysfunction, neuroinflammation, and impaired synaptic connectivity [7].

Stroke remains a leading cause of adult disability and mortality worldwide [8,9]. It is broadly classified into ischemic and hemorrhagic strokes, with ischemic stroke accounting for more than 80% of all cases [10]. The core pathological processes include energy failure, oxidative stress, excitotoxicity, inflammatory responses, and neuronal apoptosis, all triggered by the interruption of cerebral blood flow [11], ultimately resulting in neurological impairment. As the principal cellular components of the central nervous system (CNS) [12], both neurons and glial cells participate in complex molecular cascades following stroke, playing crucial roles in maintaining neural function and supporting neuroprotective and repair processes.

As a core member of the TIA family, TIA1 is a major nucleator of stress granules (SGs), and TIA1 deficiency is linked to impaired ability to form SGs [13]. TIA1 participates in key post-transcriptional regulatory processes, reflecting its broad functional repertoire in RNA metabolism [14,15,16]. Owing to its widespread expression in both neurons and glial cells [17], elucidating the cell-type-specific roles of TIA1 is essential for uncovering the pathological mechanisms of neurodegenerative diseases and stroke and may provide valuable insights for the identification of novel therapeutic targets.

## 2. Structural Features and Functions of TIA1

### 2.1. Structure of TIA1

Since its discovery, the structural characterization of TIA1 has been progressively refined, with comprehensive insights established. TIA1 is composed of three N-terminal RNA recognition motifs (RRMs) and a C-terminal domain enriched in glutamine and asparagine (Q/N-rich) [18,19]. Each RRM contains two conserved ribonucleoprotein motifs, RNP1 and RNP2, which undergo conformational changes upon specific RNA binding to stabilize an RNA–protein complex [20]. The third RRM (RRM3) plays a pivotal role in RNA binding and mediates the cytoplasmic translocation of TIA1 under stress conditions. The C-terminal Q/N-rich domain facilitates protein–protein interactions and is critical for SG formation [21]. Under steady-state conditions, TIA1 resides predominantly in the nucleus, with a minor fraction localized in the cytoplasm. Upon cellular stress, TIA1 translocates to the cytoplasm via RRM3-mediated RNA binding and participates in SG assembly [22,23].

### 2.2. Biological Functions of TIA1

TIA1 exhibits dynamic subcellular localization [24] and plays a crucial role in post-transcriptional gene regulation (Table 1 [23,25]). Under physiological conditions, TIA1 is essential for the development of T and B lymphocytes. Studies have demonstrated that *Tia1/Tial1* knockout mice exhibit severely impaired generation of antigen-specific germinal center B cells and a significant reduction in high-affinity antibody production in response to T-dependent antigens such as NP-KLH [26]. These findings underscore the necessity of TIA1 for B cell development, differentiation, and antibody production within germinal centers, highlighting its key role in humoral immunity. Furthermore, TIA1 promotes the survival of germinal center B cells by regulating Myeloid cell leukemia-1 (Mcl1) mRNA [27]. Together with TIAL1, TIA1 helps maintain timely expression of the anti-apoptotic protein Mcl1 in germinal center B cells during antigen stimulation, thereby supporting B cell survival and function and ensuring a robust germinal center response.

Under pathological conditions, TIA1 modulates translation efficiency through binding to the target mRNAs at 5′ or 3′ untranslated regions (UTRs) [28,29]. For example, during inflammatory responses, TIA1 binds to AU-rich elements (AREs) in TNF-α and COX-2 mRNAs, leading to translational repression and contributing to the containment of excessive inflammation [30]. Beyond its role in translation control, TIA1 participates in alternative splicing regulation [31,32]. It facilitates the recruitment of U1 snRNP by binding to U-rich sequences, which are typically located in intronic regions near splice sites. This splicing function is particularly important for neuron-specific splicing programs [33], including the regulation of neurofibromatosis type 1 (NF1) pre-mRNA splicing [34].

**Figure 1:**
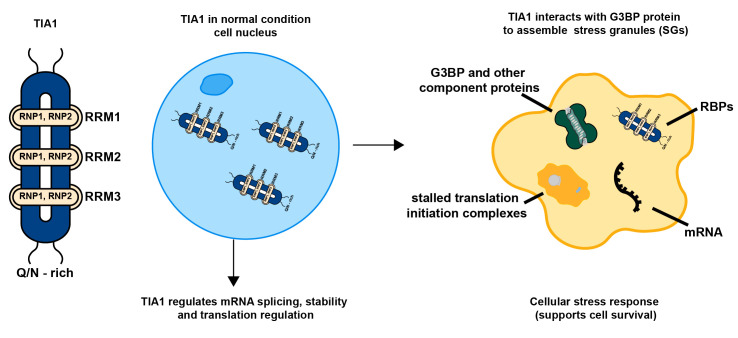
Physiological functions of TIA1 and the cellular response mechanism of TIA1 in SG assembly under cellular stress. In this diagram, the structural representation of TIA1 is shown in dark blue, the nucleus is depicted in light blue, and the cytoplasm is indicated in yellow. Under normal conditions, nuclear TIA1 is involved in mRNA splicing, stability, and translation regulation. Upon cellular stress, TIA1 interacts with cytoplasmic proteins such as G3BP, leading to the stalling of translation initiation complexes and their co-assembly into stress granules (SGs), thereby activating a pro-survival stress response program.

Notably, under conditions of oxidative stress or energy deprivation, TIA1 collaborates with proteins such as G3BP to drive the assembly of SGs [16,35]. Oxidative stress is a state of biochemical imbalance characterized by the excessive production of reactive oxygen species (ROS) and reactive nitrogen species (RNS), which overwhelms the cellular antioxidant defenses [36,37]. Under normal physiological conditions, redox homeostasis is maintained through antioxidant systems that clear ROS and RNS. However, specific stimuli such as inflammation, ischemia–hypoxia, or external injury can induce oxidative stress beyond the cellular regulatory capacity, triggering pathological processes including membrane lipid peroxidation, DNA strand breaks, and protein oxidation [38,39]. During cellular stress, kinases such as PKR and PERK become activated and phosphorylate eIF2α, preventing the formation of the eIF2-GTP-Met-tRNAi ternary complex and consequently blocking mRNA translation. TIA1 binds to adenine–uridine-rich elements (AREs) in target mRNAs via its RRM domains and facilitates the assembly of the 48S pre-initiation complex. Through its prion-like domains (PrLDs), TIA1 undergoes liquid–liquid phase separation, promoting polymerization and driving the aggregation of these complexes into SGs [40]. Simultaneously, other SG-associated proteins cooperate with TIA1 to stabilize the granule structure, which sequesters stalled translation pre-initiation complexes, thereby protecting mRNAs and inhibiting protein synthesis [41] (Figure 1). Furthermore, SG formation promotes cell survival by sequestering specific apoptosis regulators. For instance, under stress conditions, the receptor for activated C kinase 1 (RACK1) is confined within SGs, leading to inhibition of the pro-apoptotic p38 and c-Jun N-terminal kinase (JNK) pathways [35].

However, TIA1 exhibits a dual role in apoptosis regulation: it suppresses apoptosis under physiological conditions, whereas under pathological stress, excessive oxidation or mutant TIA1 promotes aberrant SG assembly, ultimately inducing cell death [42]. Additionally, the TIA1 family also significantly influences neuronal repair. For example, the *C. elegans* homolog TIAR-2 forms granules via phase separation and negatively regulates axonal regeneration both in vivo and in vitro, illustrating the functional relevance of TIA1 family proteins in neuronal repair through biomolecular condensation [43].

## 3. Expression and Function of TIA1 in Neurons

### 3.1. Expression Patterns of TIA1 in Neurons

TIA1 is widely expressed in the mammalian CNS, with high expression levels observed in the hippocampus, cortex, and spinal motor neurons [44]. Under physiological conditions, immunohistochemistry reveals that TIA1 is predominantly localized in the nucleus, where it participates in transcriptional regulation. Under endoplasmic reticulum stress, heat shock, or oxidative stress, it rapidly translocates to the cytoplasm and promotes the assembly of SGs [45]. In the cytoplasm, TIA1 is rapidly phosphorylated, promoting liquid–liquid phase separation and facilitating the recruitment of RBPs and mRNAs into SGs, thereby protecting cells from injury [16,46]. The accumulation of cytoplasmic SGs has been closely associated with neuronal death in various neurological disorders [25,47]. SG formation offers significant advantages for cell physiology, such as reducing energy consumption, regulating protein and ribostasis, and improving cell survival under adverse conditions [48]. However, impaired dynamics of SGs can accelerate neurodegeneration. When SGs fail to disassemble properly, these initially protective structures may evolve into persistent, cytotoxic aggregates. Such persistent SGs can induce the pathological aggregation of RBPs, disrupting neuronal homeostasis and ultimately leading to neuronal death [49,50].

Concurrently, TIA1 is essential for neurodevelopment. Its expression directly influences molecular targets linked to neuronal differentiation during early human brain development. During the human embryonic stem cell (hESC) stage, TIA1 binds to the broadest range of mRNA targets, many of which are key genes involved in neural development and cell differentiation. As cells differentiate from pluripotent hESCs into neural progenitor cells (NPCs) and subsequently mature neurons, the number of mRNAs bound to TIA1 decrease substantially [51].

### 3.2. Functional Regulation of TIA1 in Neurons

#### 3.2.1. Neuronal TIA1 in Neurodegenerative Diseases

**Figure 2:**
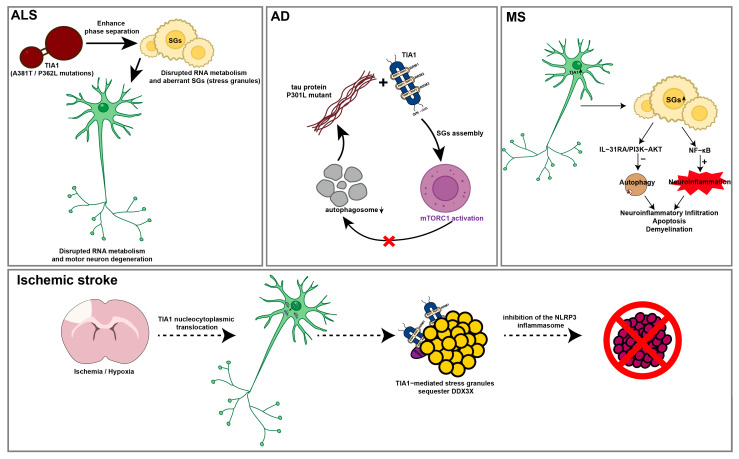
Schematic diagram of the pathological mechanisms of TIA1 in ALS, AD, MS, and ischemic stroke. In this schematic, arrows represent the progression of pathological mechanisms or the upregulation/downregulation of cellular components. In amyotrophic lateral sclerosis (ALS), where neurons are shown in green, TIA1 mutations (in dark red) enhance phase separation, leading to aberrant stress granules (in yellow) and disrupted RNA metabolism. In Alzheimer’s disease (AD), tau protein P301L mutant (in brown) binds to TIA1 (indicated by a plus sign), affecting stress granule assembly and clearance, accompanied by mTORC1 activation (in purple) and reduced autophagosomes (in gray). In multiple sclerosis (MS), the IL-31RA/PI3K-AKT pathway (inhibition indicated by a minus sign) regulates autophagy (phagocytosis in light brown) and activation of the NF-κB pathway (indicated by a plus sign) which regulates inflammation (in red), contributing to demyelination. In ischemic stroke, ischemia/hypoxia induces nucleocytoplasmic translocation of TIA1 and formation of stress granules (yellow), which sequester key factors such as DDX3X (in purple) and are associated with NLRP3 (in magenta). Across these conditions, stress granules dynamics modulate cellular stress responses and survival. A red cross symbol is used throughout to indicate inhibition.

Neurodegenerative diseases comprise a group of complex disorders characterized by the progressive degeneration of neuronal structure and function, including amyotrophic lateral sclerosis (ALS) [52], Alzheimer’s disease (AD) [53], multiple sclerosis (MS) [54], Huntington’s disease (HD) [55] and Parkinson’s disease (PD) [56], all of which constitute major threats to human health. TIA1 has recently been identified as a pivotal player in the pathophysiology of several neurodegenerative conditions (Figure 2). Genetic mutations and aberrant expression of TIA1 are associated with detrimental cellular outcomes, such as neuronal death, impaired RNA metabolism, and pathological protein aggregation.

ALS is an adult-onset neurodegenerative disease characterized by the loss of motor neurons in the brain and spinal cord [57,58], which is marked by progressive muscle weakness and atrophy, leading to paralysis and ultimately fatal respiratory failure [59]. In ALS, the A381T mutation in *Tia1* has been shown to enhance its self-assembly via inducing β-sheet interactions, resulting in the formation of irreversible amyloid fibrils and aberrant SG formation [60,61]. This disrupts RNA and protein homeostasis within neurons, contributing to motor neuron degeneration. Additionally, the P362L mutation within the low-complexity domain (LCD) of *Tia1* augments its phase separation ability, leading to abnormal SG persistence and impairing their dynamic disassembly [40]. TIA1 also acts synergistically with other RBPs, further aggravating ALS pathogenesis and underscoring its potential as a therapeutic target [40,60,62,63,64].

AD is a chronic neurodegenerative disorder and the most common form of dementia [65], characterized by neuronal death and synaptic loss in the cerebral cortex and specific subcortical regions [66,67]. Its major pathological features are the accumulation of extracellular β-amyloid plaques and intracellular neurofibrillary tangles (NFTs) [68]. NFTs are composed of abnormally phosphorylated tau protein, a process strongly linked to TIA1. In neurons from AD and other tauopathies such as FTDP-17, TIA1 significantly colocalizes with hyperphosphorylated tau, particularly within NFTs, and this association becomes more pronounced as the disease progresses [69]. Under pathological stress such as that induced by Aβ, tau translocates from axons to dendrites and recruits TIA1. Tau, especially P301L mutants, enhances TIA1 aggregation and accelerates SG formation. Within SGs, TIA1 sequesters tau, inhibits its clearance, and promotes tau misfolding, phosphorylation, and aggregation into insoluble species—ultimately leading to neurotoxic tau oligomers production [70]. In AD, abnormally accumulated tau binds directly through its N-terminus to TIA1, sequestering TIA1 in the cytoplasm, where it suppresses global protein synthesis. This results in elevated intracellular free amino acids, which activates mechanistic target of rapamycin complex 1 (mTORC1) signaling at the lysosome and suppresses autophagosome formation. Consequently, autophagy is impaired, further promoting tau accumulation through a positive feedback loop [71,72]. Additionally, TIA1-promoted tau oligomers disrupt microtubule networks, impair autophagic vesicle transport, and induce autophagic vacuole accumulation, while mutations in the valosin-containing protein (VCP) gene further exacerbate this dysfunction by directly compromising VCP-mediated clearance of SGs [73]. Together, these interconnected processes create a self-sustaining pathological loop that drives progressive neurodegenerative pathology in AD.

MS is an immune disease that induces acute and chronic inflammation in the CNS, resulting in progressive tissue damage [74], with its global prevalence continuing to rise. The disease is initiated by aberrant activation of peripheral autoreactive T cells—particularly CD4^+^ Th1 and Th17 subsets—and B cells, which cross the compromised blood–brain barrier (BBB) through the release of pro-inflammatory mediators [75]. Growing evidence suggests that neurodegenerative dysfunction in MS is closely associated with SGs [25,47]. In spinal cord neurons of EAE mice, TIA1 expression is upregulated and translocates from the nucleus to the cytoplasm. This redistribution promotes chronic SG formation, which inhibits autophagy and facilitates toxic protein aggregation. Furthermore, TIA1 activates the PI3K/AKT signaling pathway by upregulating IL-31 and its receptor, IL-31RA. This activation further inhibits autophagy, impedes SG clearance, and triggers NF-κB-mediated release of pro-inflammatory factors. Together, these events exacerbate neuroinflammatory infiltration, apoptosis, and demyelination. Notably, in vivo knockdown of TIA1 or pharmacological inhibition of PI3K disrupts the IL-31RA/PI3K/AKT/NF-κB cascade, restores autophagic flux, reduces SG accumulation, and ameliorates neurodegenerative pathology in EAE mice. These findings highlight TIA1 and its downstream pathways as promising therapeutic targets for MS [16].

HD is an autosomal dominant neurodegenerative disorder caused by an expanded CAG trinucleotide repeat in the huntingtin (HTT) gene on chromosome 4p16.3, resulting in the production of mutant HTT protein [55]. In HD, the high expression of mutant HTT in neurons induces cellular stress and interacts with key SG components such as G3BP1, triggering the formation of aberrant SGs. However, these granules become dysregulated and persistently sequester essential RNAs and proteins. This disruption impairs core RNA metabolic processes, including translation, transport, and degradation, and facilitates the transition of SGs into gel-like inclusions that accumulate over time. Such accumulation compromises cellular homeostasis and promotes neuronal apoptosis. Furthermore, a subset of long non-coding RNAs (lncRNAs) enriched within these granules, such as LHR1-LNC1610-1, is regulated by the transcriptional repressor REST. Dysregulated expression of these lncRNAs contributes to widespread transcriptomic disturbances in neurons, thereby exacerbating HD pathogenesis [76]. As a core component of SGs, TIA1 is likely to play a crucial role in this pathogenic cascade.

Alpha synuclein (αSyn) is an abundant neuronal protein normally localized to the cytosol or associated with presynaptic membranes. In PD and the related disorder dementia with Lewy bodies (DLB), αSyn aggregates to form the principal component of Lewy bodies and Lewy neurites [77]. Notably, SGs are deficient in brain samples from PD patients, and the depletion of SGs promotes α-syn aggregation [78], suggesting a protective role for SGs in PD. Thus, targeting TIA1-mediated SG formation may represent a potential therapeutic strategy to protect neurons from detrimental stress in PD.

The RNA-binding protein TIA1 serves as a critical regulator of SG assembly and plays a dual role in neurodegenerative diseases. Under physiological conditions, it is essential for neurodevelopment and crucial for promoting cell survival by forming dynamic SGs. Under pathological conditions, however, TIA1 mutations (e.g., A381T and P362L in ALS) or its pathological sequestration (e.g., by tau in AD) disrupt this equilibrium, leading to persistent SGs that transform into cytotoxic aggregates. The downstream consequences are profound and convergent: TIA1 dysfunction disrupts RNA and protein homeostasis, impairs autophagic clearance of toxic aggregates, and triggers sustained neuroinflammation. Given its central role in diseases such as AD, ALS, MS, HD and PD, TIA1 and its regulatory pathways represent a promising common therapeutic target.

#### 3.2.2. Neuronal TIA1 in Stroke Pathogenesis

Stroke, also known as cerebral stroke or cerebrovascular accident, is a severe neurological disorder. Although thrombolysis and endovascular thrombectomy are available, therapeutic options remain limited for the majority of stroke patients [79,80]; hence, it is critical and urgent to find effective preventive measures and therapeutic drug targets.

Ischemic stroke involves a series of interconnected pathological processes. Upon stroke onset, ischemia and hypoxia lead to neuronal depolarization and excessive release of the excitatory neurotransmitter glutamate. This overactivates the NMDA receptor, inducing a massive influx of calcium and resulting in intracellular calcium overload. The elevated calcium levels activate a cascade of calcium-dependent enzymes, ultimately resulting in neuronal damage [81,82]. In addition, the abrupt reduction in cerebral blood flow after ischemic stroke causes energy failure in neurons and glia, accompanied by decreased ATP production. This energy deficit further triggers membrane depolarization [83]. Mitochondrial dysfunction exacerbates energy depletion, disrupting the respiratory chain, leading to the generation of excessive reactive oxygen species (ROS) and triggering oxidative stress [84]. These processes collectively contribute to neuronal injury and inflammatory responses.

During stroke, neurons are exposed to various stressors, leading to the translocation of TIA1 from the nucleus to the cytoplasm. Through multimerization mediated by its C-terminal PrLDs, TIA1 nucleates the assembly of dynamic SGs by recruiting various RBPs and mRNA. These granules serve as critical cytoprotective structures under adverse conditions. Under physiological conditions, the SG protein DEAD-box RNA helicase 3X (DDX3X) freely interacts with the NOD-like receptor family pyrin domain containing 3 inflammasome (NLRP3) in the cytosol, a process that facilitates the oligomerization of the NLRP3 inflammasome. This assembly activates caspase-1, culminating in a robust inflammatory response and pyroptotic cell death, which worsens brain injury. However, during SG formation, TIA1 efficiently sequesters DDX3X within SGs, preventing its engagement with NLRP3 and suppressing inflammasome activation. Early remote ischemic conditioning (RIC) enhances this protective process by boosting SG assembly, further reducing free DDX3X levels, suppressing the NLRP3 inflammasome, and ultimately attenuating neuronal pyroptosis and damage after stroke [85] (Figure 2). Concurrently, studies indicate that TIA1-mediated SG formation protects against neuronal apoptosis in ischemic stroke, involving pathways such as p38 MAPK and JNK [86].

In summary, TIA1 plays a neuroprotective role in ischemic stroke by orchestrating SG formation in response to ischemic stress. Its translocation from the nucleus to the cytoplasm initiates the assembly of SGs, which sequester pro-inflammatory factors such as DDX3X. This sequestration critically inhibits DDX3X-mediated activation of the NLRP3 inflammasome, thereby attenuating caspase-1-driven pyroptosis and inflammatory injury. Additionally, TIA1 may further safeguard neurons by modulating apoptosis-related pathways, including p38 MAPK and JNK. As research advances, targeted intervention strategies directed at TIA1 hold considerable promise and may become integral to comprehensive stroke management, potentially improving neurological outcomes and long-term prognosis (Table 2).

## 4. Expression and Function of TIA1 in Glial Cells

### 4.1. Expression Patterns in Glial Cells

TIA1 is widely expressed in CNS glial cells, including astrocytes, microglia, and oligodendrocytes. Under normal physiological conditions, TIA1 is predominantly localized in the cytoplasm. Similarly to its expression in neurons, TIA1 localization in glial cells is dynamic and regulated by various stimuli, such as oxidative stress and inflammatory factors [35]. As a key nucleating protein of SGs, TIA1 modulates SG assembly by binding to stalled translation initiation complexes. Under oxidative stress, oxidative modification of TIA1 affects SG formation, leading to the accumulation of misfolded proteins and triggering the apoptotic pathways [35].

Astrocytes, the most abundant type of glial cells in the CNS, are essential for maintaining the BBB, controlling synaptic activity, and providing metabolic support. In astrocytes, TIA1 expression exhibits a notable state-dependent profile, especially during the transition from resting to reactive states. Single-cell transcriptomic analysis has revealed that astrocytes undergo dynamic phenotypic remodeling in response to inflammatory stimuli, initially adopting a neuroprotective substate characterized by elevated expression of heat shock proteins and metabolism-related genes, which later transforms into a neurotoxic substate with upregulation of pro-inflammatory factors and complement components. This progression indicates that upon exposure to adverse external stimuli, astrocytes initially exert a protective effect toward neurons but eventually shift into a detrimental state over time. This transition is closely associated with alterations in TIA1 expression and SG formation, underscoring TIA1’s pivotal role in regulating astrocyte function [87].

TIA1 is also expressed in microglia. Under physiological conditions, it is predominantly localized to the nucleus, where it participates in mRNA splicing and transport, maintaining microglial homeostasis and preventing excessive inflammatory responses [88]. In neuroinflammatory and neurodegenerative disorders, microglia, as the resident innate immune cells of the CNS, display significant phenotypic plasticity.

Similarly to the other glial cell types, TIA1 in oligodendrocytes is primarily localized to the nucleus under normal conditions, but rapidly translocates to the cytoplasm upon cellular stress, where it participates in the assembly of SGs [89].

### 4.2. Functional Regulation of TIA1 in Glial Cells

#### 4.2.1. TIA1 Expression in Glial Cell in Neurodegenerative Diseases

Under sustained stress conditions in AD, including elevated glucocorticoids, oxidative stress, and inflammatory mediators, astrocytes exhibit marked pathological alterations. Tau undergoes pathological hyperphosphorylation and colocalizes with TIA1 in the astrocytic cytoplasm, accelerating the conversion of soluble tau monomers into insoluble fibrils and promoting the formation of neurotoxic aggregates. Furthermore, TIA1-positive SGs sequester key autophagy adaptor proteins, disrupting the autophagosome–lysosome pathway and preventing tau clearance. These TIA1–tau complexes are subsequently co-deposited within glial inclusions. Chronic stress also activates the mTOR pathway in astrocytes, which inhibits the critical autophagy initiator ULK1 (UNC-51-like kinase 1), thereby preventing SG disassembly and promoting their maturation into solid, persistent aggregates. This cascade further compromises cellular recovery mechanisms [87,90]. Sustained TIA1 aggregation further promotes neuroinflammation by driving astrocytes and microglia into neurotoxic subtypes. This shift in turn stimulates NLRP3 inflammasome formation and the release of pro-inflammatory factors such as IL-1β and TNF-α, which severely damage the BBB and facilitate the infiltration of peripheral immune cells, inducing a chronic neuroinflammatory microenvironment to accelerate disease progression [91].

As intrinsic immune cells of the CNS, microglia are responsible for clearing misfolded proteins (such as Aβ and tau), cellular debris, and apoptotic cells to maintain brain homeostasis, whose functional state is precisely regulated by TIA1. Under physiological conditions, TIA1 contributes to microglia homeostasis, whereas under pathological stress, it promotes SG formation and modulates the magnitude and duration of immune responses. In the MS model of EAE mice, TIA1 expression in microglia was markedly increased, and the expression ectopically translocated from the nucleus to the cytoplasm to assemble SGs. Studies have demonstrated that targeted suppression of TIA1 in microglia dramatically attenuates neuroinflammation, demyelination, and axonal damage, while improving motor impairment in EAE mice [16]. TIA1 also influences microglial immune synaptic function by regulating the stability of mRNAs encoding cytokines such as TNF-α and IL-1β [92]. Under hypoxic–ischemic injury, elevated TIA1 expression enhances NLRP3 inflammasome activation, facilitating the maturation and release of IL-1β to exacerbate neuronal damage [93]. Further research has revealed that TIA1 expression in microglia is closely associated with tau pathology-induced neuroinflammatory responses. In P301S mice, microglia exhibited significant activation of sensome genes (e.g., TREM2 and CLEC7A), complement genes (e.g., C1q family), and inflammation-related genes (e.g., TNFα and IL-1β). These cells adopt an activated amoeboid morphology with reduced cell size and complexity, accompanied by increased CD68^+^ staining. In *Tia1* knockout mice, activation of these inflammatory pathways was markedly suppressed. Microglia maintained a resting branched morphology, and expression levels of these genes approached those of wild-type controls [88]. Notably, TIA1 deficiency in microglia not only suppressed neuroinflammation but also markedly attenuated tau pathology progression. At 9 months of age, *Tia1* knockout mice exhibited lower levels of misfolded tau, phosphorylated tau, and fibrillar tau in the hippocampal CA1 and CA3 regions and the entorhinal cortex compared to P301S mice, indicating that TIA1 accelerates tau aggregation and neurodegenerative disease progression [88]. Additionally, TIA1 impairs microglial phagocytosis through the sequestration of spleen tyrosine kinase (SYK), a key signaling molecule downstream of phagocytic receptors such as TREM2. Upon stimulation by Aβ oligomers, SYK dissociates from membrane receptors and becomes trapped within TIA1-positive SGs, disrupting phagocytic signaling and compromising clearance capacity [94].

Oligodendrocytes, the myelinating cells of the CNS, are indispensable for the formation and maintenance of myelin sheaths. Their high metabolic demand for myelin synthesis, particularly their reliance on glycolysis, renders them highly susceptible to metabolic and oxidative stress. In MS, SGs have been identified in oligodendrocytes residing within both lesional and normal-appearing white matter. Acute oxidative stress triggers phosphorylation of eIF2α and activates the integrated stress response, which, often in concert with pro-inflammatory factors, induces the formation of persistent SGs. As a core SG component, TIA1 contributes to oligodendrocyte dysfunction and myelination deficits by disrupting RNA metabolism and promoting aberrant localization of RBPs [95]. As MS progresses, the chronic inflammation continues to activate microglia, which drives axonal damage, neuronal degeneration, and ferroptosis [96,97]. Concurrently, inhibitory signals from the inflammatory milieu impair the differentiation capacity of oligodendrocyte precursor cells and hinder remyelination, thereby promoting glial scar formation. These pathological processes collectively contribute to irreversible cerebral atrophy, axonal loss, and permanent neurological deficits [98,99]. In the context of ALS and frontotemporal dementia (FTD), mutations in the *Tia1* gene or prolonged stress are proposed to promote SG formation, which undergo a pathogenic transition from dynamic structures into insoluble aggregates. This aberrant phase transition is hypothesized to autonomously disrupt local mRNA translation, compromising the ability of oligodendrocytes to maintain myelin integrity. Moreover, these pathological granules are implicated in the sequestration of key RBPs like TDP-43, thereby initiating a self-reinforcing cascade in which protein mislocalization and pathological aggregation drive each other forward [89].

**Figure 3:**
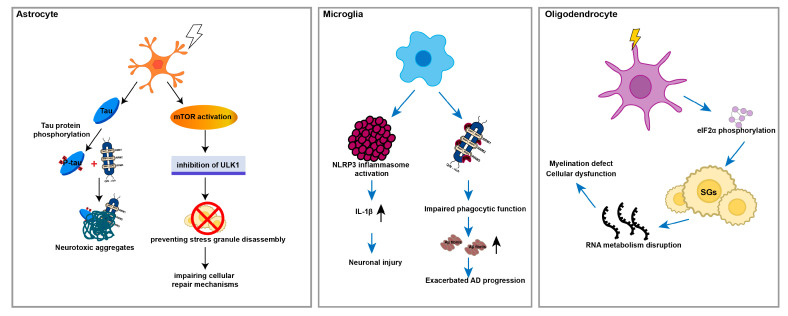
The pathological roles of TIA1 -mediated stress granules across three major glial cell types in neurodegenerative disorders. In this schematic, the three glial cells are distinguished by color, astrocytes in orange, microglia in blue, and oligodendrocytes in purple. Within the astrocyte section, dark green represents neurotoxic aggregates formed by p-Tau and TIA1. The lightning bolt symbol denotes cellular stress. Arrows indicate the causal relationships or progression of pathological mechanisms within each cell type. In astrocytes, tau pathology inhibits ULK1, thereby preventing the normal disassembly of TIA1-associated stress granules and impairing cellular repair. In microglia, NLRP3 inflammasome activation and IL-1β release contribute to neuronal injury, processes potentially linked to TIA1 dysregulation. In oligodendrocytes, disruption of RNA metabolism and cellular dysfunction directly impact TIA1-mediated stress granule homeostasis, leading to myelination defects. These mechanisms collectively highlight the central role of TIA1 in glia-driven neurodegeneration.

In summary, TIA1 acts as a critical node linking glial stress responses, RNA metabolism, and neuroinflammation in neurodegenerative conditions. Its pathological involvement includes promoting protein aggregation, sustaining inflammatory signaling, and compromising glial support functions (Figure 3). As it plays a multifaceted role in glial cells and significantly contributes to the pathogenesis of some neurodegenerative disorders, targeting TIA1 dynamics and its associated pathways may offer novel therapeutic strategies for modulating glial reactivity and slowing disease progression. Although no current evidence directly links TIA1 expression in glial cells to HTT or PD, the established role of SG pathogenesis in both disorders [100,101] suggests that future investigations may reveal such a connection.

#### 4.2.2. TIA1 Expression in Glial Cells in Stroke

Under normal physiological conditions, glial cells provide essential structural and metabolic support for CNS development [102]. Following stroke, microglia and astrocytes actively participate in the regulation of neuroinflammation [103,104,105].

Astrocytes also respond dynamically to ischemic stroke. Under ischemic or hypoxic stress, TIA1 is rapidly recruited into SGs, leading to the suppression of non-essential protein synthesis. Beyond this stress response, astrocytes support neurological recovery by promoting angiogenesis, neurogenesis, synaptogenesis, and axonal remodeling [106]. They further provide neuroprotection through anti-excitotoxic mechanisms as well as the secretion of neurotrophic factors [107].

At the ischemic site, microglia respond rapidly to ischemia. During the acute phase, they modulate neuroinflammatory processes through the release of various inflammatory mediators [108]. Then, TIA1 expression is markedly upregulated in microglia. This upregulation promotes the assembly of SGs, which sequester *Igf2* mRNA and suppress its translation. Conditional ablation of *Tia1* specifically in microglia impairs SG formation, leading to increased IGF2 expression. The elevated IGF2 signaling subsequently drives microglial polarization toward an anti-inflammatory phenotype, enhances phagocytic clearance of cellular debris, and ultimately mitigates neuroinflammation and neuronal loss, thereby improving functional recovery [109]. As the ischemic injury progresses beyond the acute phase, the inflammatory response gradually subsides. During this transition, microglia form SGs with TIA1 as a key component and shift toward a reparative phenotype [110]. In addition, microglia contribute to tissue recovery by influencing synaptic activity through the regulation of cell migration and synaptic pruning in the CNS [111].

Oligodendrocytes are a key type of glial cell in the CNS. Their high energy demands during axonal myelination, elevated intracellular iron levels, and low reduced glutathione render them particularly susceptible to ischemic damage. This damage contributes to demyelination and axonal destabilization, leading to white matter injury and long-term neurological deficits. Stroke-induced oxidative stress and intracellular calcium overload can directly trigger oligodendrocyte apoptosis or necrosis, thereby exacerbating white matter injury and hindering recovery. In the post-ischemic stroke phase, oligodendrocytes and their precursor cells may exert neuroprotection through the secretion of neurotrophic factors, supporting neuronal survival and repair. Via cytokine release and crosstalk with microglia, oligodendrocytes participate in the cerebral immune and inflammatory responses, while moderate inflammation aids dead cell clearance and tissue repair [112]. However, studies specifically examining TIA1 expression in oligodendrocytes following stroke are currently lacking. Given the established relevance of SGs to stroke and the significant role of oligodendrocytes in its pathogenesis, breakthroughs in this area could consequently offer important new perspectives for understanding stroke mechanisms and developing treatments.

TIA1 is closely associated with the pathogenesis and neural recovery processes following stroke, including SG formation, mRNA stability, inflammatory response, and apoptosis, all of which play critical roles in stroke injury progression and repair. Under acute ischemic–hypoxic stress, TIA1 drives the formation of SGs, which act as protective hubs. These SGs globally suppress non-essential protein translation to conserve energy, while TIA1 specifically sequesters mRNAs encoding detrimental proteins such as pro-apoptotic factor Bax and inflammatory cytokines (TNF-α and IL-6), thereby mitigating their harmful effects. Concurrently, this process facilitates the selective translation of survival-promoting factors such as brain-derived neurotrophic factor (BDNF). Thus, TIA1 and SGs serve a critical dual role: their transient formation promotes cell survival, whereas persistent SG assembly can shift the balance towards apoptosis. Given its central position at the crossroads of multiple injury pathways, TIA1 presents a significant therapeutic target (Table 3). Future research exploring its role in BBB dysfunction and tissue-specific functions may pave the way for novel precision treatments of stroke.

## 5. Summary and Discussion

In this review, we have systematically outlined the structural characteristics and functions of TIA1, as well as its expression profile in the nervous system and its therapeutic potential in neurodegenerative diseases and stroke. As a core regulator of RNA metabolism, TIA1 is expressed in both neurons and glial cells.

Under physiological conditions, TIA1 facilitates neurodevelopment and cell survival through dynamic SG formation. In neurodegenerative diseases, however, genetic mutations (e.g., A381T and P362L in ALS) or pathological interactions (e.g., with tau in AD and mutant HTT in HD) disrupt its normal function in neurons. This leads to the formation of persistent, aberrant SGs that transform into cytotoxic aggregates. The core pathological consequences converge on disrupted RNA and protein homeostasis, impaired autophagy, and sustained neuroinflammation, which are common features across ALS, AD, MS, HD, and PD. In glial cells, TIA1 further exacerbates disease pathology by driving aberrant SG assembly. It promotes tau aggregation and impairs autophagy in astrocytes, drives microglia toward a pro-inflammatory phenotype while compromising their phagocytic capacity, and disrupts RNA metabolism and myelin maintenance in oligodendrocytes.

During ischemic stroke, neuronal TIA1 sequesters DDX3X within SGs, preventing its interaction with the NLRP3 inflammasome and thereby inhibiting inflammasome activation, pyroptosis, and subsequent neuronal injury. Additionally, TIA1-mediated SG formation protects against apoptosis through pathways involving p38 MAPK and JNK. TIA1 expression also modulates glial responses to injury and repair. In microglia, upregulated TIA1 promotes SG formation, which sequesters *Igf2* mRNA and helps regulate neuroinflammatory signaling. In astrocytes, TIA1 is recruited into SGs to suppress global protein synthesis while simultaneously supporting neuroprotective functions.

TIA1 plays pivotal roles in regulating apoptosis, inflammation, oxidative stress, and key signaling pathways, thereby representing a multidimensional therapeutic target. The field nevertheless continues to evolve rapidly, with several critical issues remaining unresolved. First, the mechanism underlying the shift of TIA1-mediated SGs from a protective to a toxic state is not fully elucidated, particularly in terms of their specific contributions to autophagic dysregulation, inflammatory amplification, and loss of proteostasis. Second, defects in SG assembly caused by TIA1 deletion increase cellular sensitivity to stress, and supplementing TIA1 may enhance cell resilience. However, it remains unclear whether pharmacologically inducing SG assembly or supplementing TIA1 via gene therapy might lead to the accumulation of pathological proteins. Third, most current evidence originates from animal models and cellular experiments; the expression profile of TIA1 in human tissues and its correlation with clinical manifestations require validation in large-scale patient cohorts. Accordingly, future research should prioritize the development of targeted strategies to precisely modulate TIA1 function or disrupt its pathological aggregation, for example, through small-molecule compounds, oligonucleotide-based approaches, or gene-editing techniques. Concurrently, exploring the potential of TIA1 as a biomarker for disease staging or prognosis evaluation holds significant promise. It is expected to provide novel targets and a theoretical foundation for the precise treatment of neurodegenerative disorders and stroke, ultimately offering new avenues for therapeutic intervention.

## Figures and Tables

**Table 1:** Structure and core biological functions of TIA1.

Category	Description	Key Pathways/Molecules
Domains	Three N-terminal RRMs, C-terminal Q/N-rich domain	RRM1-RRM3, Q/N-rich domain
Post-transcriptional regulation	Regulate mRNA splicing, stability, translation via AREs/U-rich elements	AREs, U1 snRNP, TNF-α, COX-2
Stress granule assembly	Translocate to cytoplasm under stress, undergo LLPS via PrLD, nucleate SG formation	eIF2α phosphorylation, G3BP, PrLD
Immune regulation	Promote germinal center B cell survival by regulating Mcl1 mRNA	Mcl1, T-dependent antigens
Apoptosis regulation	Suppress apoptosis under physiology; promote cell death via persistent SGs under pathology	RACK1, p38/JNK pathways, Bax

**Table 2:** Expression and function of TIA1 in neurons (by disease).

Disease	Expression/Functional Role	Key Pathways/Molecules	Affected Brain Regions	Species
ALS	*Tia1* mutations (A381T/P362L) enhance self-assembly/phase separation, driving persistent SGs and amyloid fibril formation, which disrupts RNA/protein homeostasis in motor neurons.	A381T, P362L, LCD, β-sheet interactions, SGs, amyloid fibrils, RBPs	Motor cortex of the brain, motor neurons in the spinal cord	Human, Mouse
AD	Colocalize with hyperphosphorylated tau in SGs; promote tau aggregation and inhibit autophagy.	Hyperphosphorylated tau, NFTs, ULK1/2, mTORC1, VCP, autophagy	Cerebral cortex, hippocampus, amygdala, entorhinal cortex	Human, Mouse
MS	Induce chronic SGs. Activate IL-31/PI3K/AKT/NF-κB pathway to inhibit autophagy and exacerbate neuroinflammation.	IL-31RA, PI3K/AKT, NF-κB, autophagy	Spinal cord, cerebral white matter, optic nerve	Human, Mouse
HD	Mediate RNA metabolism disruption and lncRNA dysregulation, leading to neuronal transcriptomic disturbance and apoptosis.	Mutant HTT, G3BP1, lncRNAs (e.g., LHR1-LNC1610-1), REST	Striatum, cerebral cortex	HEK293 cells
PD	Assemble protective SGs against α-syn neurotoxicity.	SG assembly, α-synuclein (αSyn), Lewy bodies/neurites	Substantia nigra pars compacta, striatum, locus coeruleus	Human, HeLa cells
Stroke	Sequester DDX3X to inhibit NLRP3 inflammasome activation, modulate p38 MAPK/JNK to suppress apoptosis.	SG assembly, DDX3X sequestration, NLRP3 inflammasome, p38 MAPK/JNK	Middle cerebral artery territory, hippocampus	Rat

**Table 3:** Expression and function of TIA1 and related pathways in glial cells (by cell type).

Cell Type	Expression/Functional Role	Key Pathways/Molecules
Astrocytes	Under stress, TIA1 promotes tau aggregation, inhibits autophagy via mTOR/ULK1, and activates neurotoxic subtypes.	Tau, mTOR, ULK1, autophagy, NLRP3 inflammasome
Microglia	Under pathological stress, TIA1 promotes SGs formation, regulates cytokine mRNA stability, enhances NLRP3 inflammasome activation, and impairs phagocytosis by sequestering *SYK* or *Igf2* in SGs.	NLRP3, IL-1β, SYK, TREM2, phagocytosis signaling
Oligodendrocytes	TIA1-positive SGs disrupt RNA metabolism and impair myelination; persistent SGs contribute to oligodendrocyte dysfunction under oxidative stress.	eIF2α phosphorylation, ISR, Mbp mRNA, oxidative stress

## Data Availability

No new data were created or analyzed in this study. Data sharing is not applicable to this article.
